# A triplex quantitative real-time PCR assay for differential detection of human adenovirus serotypes 2, 3 and 7

**DOI:** 10.1186/s12985-018-0983-x

**Published:** 2018-05-02

**Authors:** Fang-zhou Qiu, Xin-xin Shen, Meng-chuan Zhao, Li Zhao, Su-xia Duan, Chen Chen, Ju-Ju Qi, Gui-xia Li, Le Wang, Zhi-shan Feng, Xue-jun Ma

**Affiliations:** 1 Hebei Medical University, Shijiazhuang, 050031 Hebei China; 2 Key Laboratory for Medical Virology, National Health and Family Planning Commission, National Institute for Viral Disease Control and Prevention, Chinese Center for Disease Control and Prevention, No. 155 Changbai Street, Chang ping District, Beijing, 102206 China; 3 Children’s Hospital of Hebei Province, Shijiazhuang, 050031 Hebei China

**Keywords:** Pneumonia, HAdV, Triplex quantitative real-time PCR, Clinical

## Abstract

**Background:**

Human adenovirus (HAdV) serotypes 2, 3 and 7 are more prevalent than other serotypes and have been associated with severe pneumonia in pediatric children. Molecular typing of HAdV is not routinely performed in clinical diagnostic laboratories as it is time-consuming and labor-intensive.

**Methods:**

In the present study, we developed a triplex quantitative real-time PCR assay (tq-PCR) in a single closed tube for differential detection and quantitative analysis of HAdV serotypes 2, 3 and 7. The sensitivity, specificity, reproducibility and clinical performance of tq-PCR were evaluated.

**Results:**

The analytical sensitivity of the tq-PCR was 100 copies/reaction for each of HAdV serotypes 2, 3 and 7, and no cross-reaction with other common respiratory viruses or HAdV serotypes 1,4,5,6,31,55 and 57 was observed. The coefficients of variation (CV) of intra-assay and inter-assay were between 0.6% to 3.6%. Of 138 previously-defined HAdV-positive nasopharyngeal aspirates samples tested, the detection agreement between tq-PCR and nested PCR was 96.38% (133/138).

**Conclusion:**

The proposed tq-PCR assay is a sensitive, specific and reproducible method and has the potential for clinical use in the rapid and differential detection and quantitation of HAdV serotypes 2, 3 and 7.

## Background

Human adenoviruses (HAdV) are nonenveloped icosahedral double-stranded DNA virus which belongs to the *Mastadenovirus* genus and classified as 7 HAdV species (HAdV-A to -G) including more than 64 serotypes [1]. Clinical mild infections associated with HAdV includes fever, acute respiratory illness, gastroenteritis and conjunctivitis. Rare manifestations of HAdV infections are hemorrhagic cystitis, hepatitis, hemorrhagic colitis, pancreatitis, nephritis, meningoencephalitis and death [2]. Different HAdV serotypes have been associated with distinct clinical syndromes [3]. HAdV are one of the major pathogens associated with febrile respiratory illness in children [4]. And acute respiratory infections (ARI) are mainly caused by HAdV species B (3, 7, 14, 21, 55), C (1, 2, 5, 6) and E (4) worldwide [5]. Previous studies reported that HAdV 2, 3 and 7 were more prevalent and have been associated with severe pneumonia in China [3, 6–9].

HAdV pneumonia in pediatric patients can progress rapidly to multi-organ failure. Due to the lack of reliable and practical methods for HAdV typing by clinical laboratories, children with HAdV pneumonia may be misdiagnosed and inadequately treated. Although no antiviral drug has been approved to treat adenovirus pneumonia, accurate and prompt detection and typing of adenovirus is highly in demand to guide antiviral treatment, reduce the disease severity [3, 10] and contribute in the monitoring of outbreaks and dynamic assessment of viral loads in transplant patients [11].

The aim of this study is to develop a triplex quantitative real time PCR (tq-PCR) assay for rapid and differential detection of HAdV 2, 3 and 7 for potential clinical use, and investigate the prevalence of HAdV infection in Hebei province, China from June to November.

## Methods

### Clinical samples

Clinical samples used in this study were collected from 200 inpatients presenting with acute respiratory symptoms at the Children’s Hospital of Hebei Province (China) between June and November, 2017. These specimens had been previously tested by the Respiratory Pathogen 13 Detection Kit [12] and 138 were found to be positive for HAdV and 62 were positive for some other respiratory virus. The clinical samples were nasopharyngeal aspirates and stored at − 80 °C until extraction of nucleic acid. And the study was conducted with the approval of the Ethics Committee of Children’s hospital of Hebei Province, and written informed consents were obtained from the children’s parents.

### Nucleic acid extraction

Total 200μL of each clinical sample was treated with Master Pure Complete DNA and RNA purification kit (Epicenter Technologies, Madison, WI) according to the manufacturer’s instructions. The extracts were eluted in 50μL of DNase- and RNase-free water and stored at− 80 °C until use.

### Primers and probes design

Both complete and partial genomes of hexon gene were derived from GenBank databases. The sequences were aligned using Vector NTI. The forward primer of this study was derived from previously published [13] and the reverse primer, three MGB probes were newly designed from hexon gene sequences using oligo7. The primers were analyzed carefully to minimize primer-primer interactions, dimer formation among themselves and the formation of secondary structures in the multiplex PCR. The fluorescent reporter dyes for type 2, 3, 7 probes were FAM, HEX, and Cy5, respectively. The primer and probes sequences are outlined in Table 1.

**Table 1:** The primer and probe of tq-pcr and nested pcr

	Primer (Probe)	Sequence (5′-3′)	GC content (%)	Tm (°C)	Reference
tq-PCR	F-primer	GGYCCYAGYTTYAARCCCTAYTC	39.13%	54.9 °C	[13] This study
	R-primer	AAYTTGAGGYTCTGGYTGATCKG	39.13%	55.7 °C	
	Probe2	FAM-TGTGAGTGGGAACAAACCGAAG-MGB	50.00%	60.6 °C	
	Probe3	HEX-ACAATGCAGTAACTACCACCACAA-MGB	41.67%	59.6 °C	
	Probe7	Cy5-TTACTGCAGACAACAAGCCCAT-MGB	45.45%	59.7 °C	
Nested PCR	AdhexF1	TICTTTGAC ATICGIGGIGTICTIGA	38.46%	60.3 °C	[13]
	AdhexR1	CTGTCIACIGCCTGRTTC CACA	45.45%	59.8 °C	
	AdhexF2	GGYCCYAGYTTYAARCCCTAYTC	39.13%	54.9 °C	
	AdhexR2	GGTTCTGTCICCCAGAGARTCIAGCA	50.00%	63.3 °C	

### Preparation of DNA standards and standard curves

PCR products of HAdV were obtained with the two step nested PCR as described in previous study [14]. The recombinant plasmids harboring the sequences of HAdV serotypes 2, 3 and 7 were constructed, respectively, and the insert size of each targeted sequence was 820 base pair (bp). Sequencing and cloning were done by TsingKe Biotech Corp (Beijing, China). The recombinant plasmids were used as standards for the quantitative analysis of tq-PCR. The plasmids were serial 10-fold diluted from 10^8^ to 10^1^ copy/μL and stored − 20 °C until use. The tq-PCR standard curves were individually generated for HAdV serotypes 2, 3 and 7 by serial 10-fold dilutions of the three recombinant plasmids with a known copy number from10^1^ to 10^8^ copies/μL, and the dilutions were quantified by Nanodrop (NanoPhotometer N60, Germany).

### The q-PCR and tq-PCR assays

The mono q-PCR assay was performed in the CFX96TM real-time system (BIORAD, USA) using Premix Ex Taq™ (Probe qPCR), ROX plus (Takara, Dalian, China). PCR amplification was initially performed in 20 μL reaction volume containing 10 μL of reaction mixture (TaKaRa Ex Taq HS, dNTP Mixture, Mg2+, Tli RNaseH and ROX Reference Dye), 0.8 μL of each of 20 μM forward primer and reverse primer, 0.4 μL of each of 10 μM type 2,3 and 7 of HAdV probes, 2 μL of total nucleic acid extracts, and 5.2 μL of RNase-free water. The thermal cycling condition was as follows: one cycle of 20s at 95 °C; 40 cycles of 1 s at 95 °C, 20s at 55 °C. Fluorescent signals were detected at the end of each cycle and the cycle threshold (Ct) value ≤38 was considered positive. The tq-PCR assay for the differential detection of HAdV serotypes 2, 3 and 7 in one tube was performed in a volume of 20μLcontaining 10 μL of reaction mixture, 0.8 μL of each of 20 μM forward primer and reverse primer, 0.4 μL of each of 10 μM type 2,3 and 7 of HAdV probes, 1 μL of each of plasmid mixture, and 4.2 μL of RNase-free water. The thermal cycling condition was unchanged.

### Analytical sensitivity, specificity and reproducibility of tq-PCR assay

The analytical sensitivity analysis of tq-PCR assay was carried out using 10-fold dilutions of plasmid range from 10^1^ to 10^8^ copies/μL and the specificity was evaluated by using 62 other common respiratory viruses-positive samples retrospectively tested by Respiratory Pathogen 13 Detection Kit (13× kit) [15]. The intra-assay coefficients of variation of this assay was tested using three plasmids in three replicates and inter-assay reproducibility was tested in three different days within a week.

### Comparison of clinical performance between the tq-PCR assay and two step nested PCR assay

A total of 138 HAdV-positive clinical samples from Children’s hospital of Hebei Province (China) were detected with the tq-PCR assay. For comparison, two step nested PCR was performed in parallel on these samples. The nested PCR products of all the 138 samples were sequenced to confirm the results of tq-PCR assay.

## Results

### Sensitivity

**Fig. 1:**
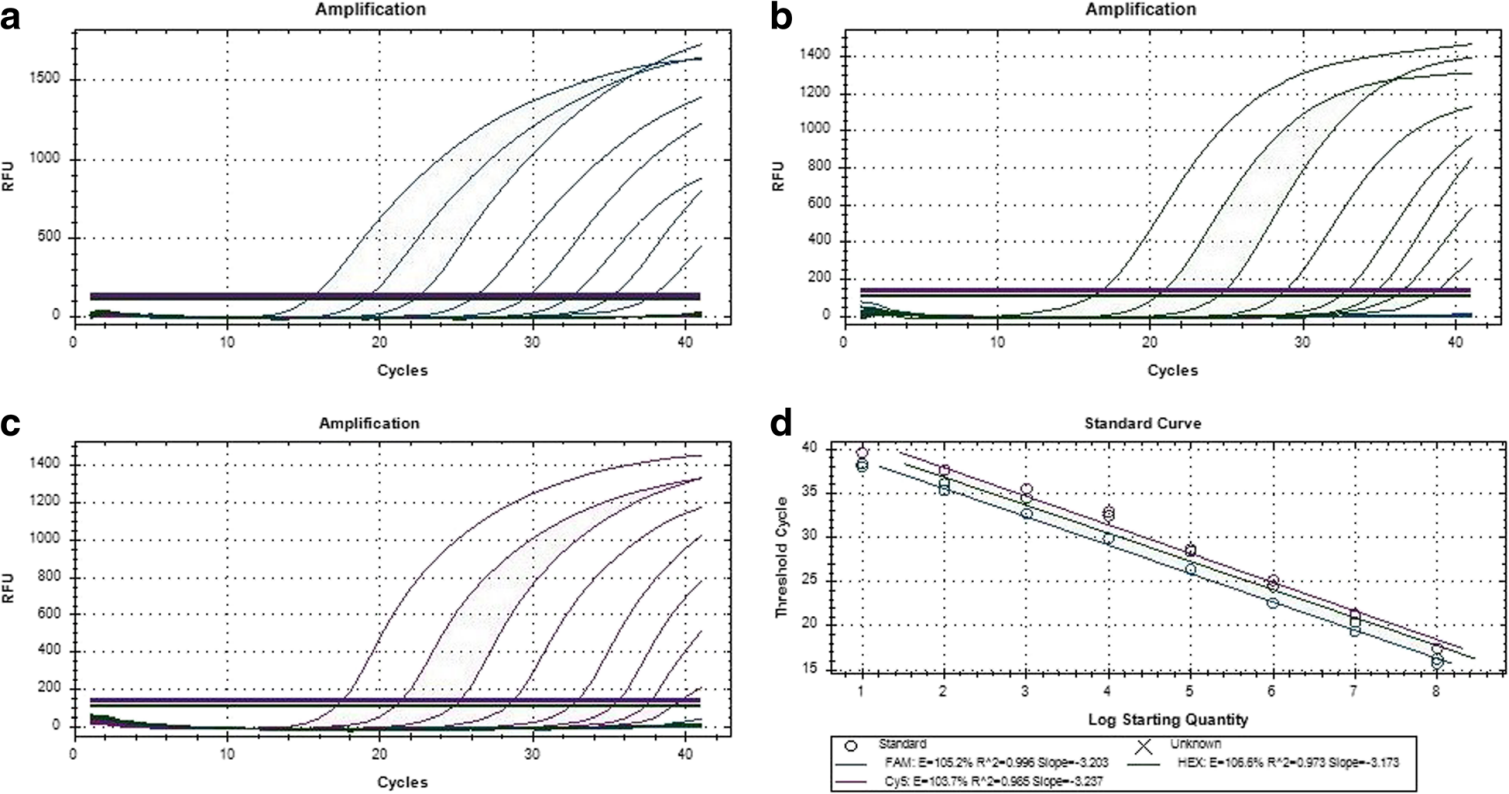
**a**, **b**, and **c** are amplification curves of the HAdV serotypes 2, 3 and 7, respectively using serial 10-fold dilutions of the three recombinant plasmids from 10^8^to 10^1^copy/μl, and **d** is the standard curves of the tq-PCR using single template individually. The correlation coefficient (R^2^) of HAdV serotypes 2, 3 and 7 were 0.996, 0.973, 0.985, respectively and amplification efficiencies for the different targets were 105.2%, 106.6% and 103.7%, respectively

The analytical sensitivity of the tq-PCR assay for each of human adenovirus 2, 3 and 7 was approximately 10^2^ copies/reaction (Fig. 1, Table 2). The standard curves of the tq-PCR assay were generated. As is shown Fig. 1d, the standard curves were linear in the range of 10^8^–10^2^ copies for the HAdV serotypes 2, 3 and 7. The correlation coefficient (R^2^) of HAdV serotypes 2, 3 and 7 were 0.996, 0.973, 0.985, respectively and amplification efficiencies for the different targets were 105.2%, 106.6% and 103.7%, respectively. Then we took plasmids of HAdV serotypes 2, 3 and 7 into one tube to simulate co-infection, a analytical sensitivity of approximately 10^3^ copies/reaction of each of these three plasmids was obtained.

**Table 2:** detection limit of tq-PCR

Copies/reaction	Ad2	Ad3	Ad7
10^1^ copy/μL	17/20	5/20	3/20
10^2^ copy/μL	20/20	20/20	20/20
10^3^ copy/μL	20/20	20/20	20/20

### Specificity

A total of 62 samples positive for other common respiratory pathogens were used to test the tq-PCR assay specificity. These pathogens included *rhinovirus, parainfluenza virus, human bocavirus, coronavirus, influenza A and B viruses, human metapneumovirus, Mycoplasma pneumonia and Chlamydophila*. The tq-PCR assay showed no cross-reactions with these pathogens (data not shown). Other HAdV species A (31), B (55), C (1,5,6), E (4) from enrolled HAdV-positive samples were also tested, and no positive results were obtained.

### Reproducibility

In order to determine the intra-assay variability of the standard plasmids, three 100-fold dilutions from 10^7^-10^3^copies/μL of HAdV serotypes 2, 3 and 7 plasmids were detected three times within the same run, the coefficient of variation (CV) that was obtained ranged from0.6 to 3.6 for all the plasmids (Table 3). The inter-assay variability was evaluated by testing three dilutions of plasmids (10^7^-10^3^copies/μL) on three different days with a week and gave a CV ranging from 1.0 to 3.6 for all the viral concentrations detected (Table 4).

**Table 3:** Intra-assay of coefficient of variation (CV) of the tq-PCR

	10^7^copy/μl			10^5^copy/μl			10^3^copy/μl		
	Ad2	Ad3	Ad7	Ad2	Ad3	Ad7	Ad2	Ad3	Ad7
CT value	17.74	20.12	20.43	24.95	26.39	26.39	32.15	33.11	35.23
	17.40	19.05	20.88	24.24	26.44	27.05	33.08	33.96	34.85
	18.34	19.56	20.29	26.05	26.13	27.31	32.59	34.52	34.53
Mean	17.83	19.58	20.53	25.08	26.32	26.92	32.61	33.86	34.87
CV%	2.7%	2.7%	1.5%	3.6%	0.6%	1.8%	1.4%	2.1%	1.0%

**Table 4:** Inter assay of coefficient of variation (CV) of the tq-PCR

	10^7^copy/μl			10^5^copy/μl			10^3^copy/μl		
	Ad2	Ad3	Ad7	Ad2	Ad3	Ad7	Ad2	Ad3	Ad7
CT value	17.52	20.56	21.23	25.59	26.33	27.23	32.78	33.45	35.12
	18.20	21.05	20.42	25.24	27.48	27.14	33.15	34.15	34.01
	18.83	19.97	20.79	26.75	26.71	27.88	32.47	34.41	34.34
Mean	18.18	20.53	20.81	25.86	26.84	27.42	32.80	34.00	34.49
CV%	3.6%	2.6%	2.0%	3.1%	2.2%	1.5%	1.0%	1.5%	1.7%

### Agreement between the tq-PCR and two step nested PCR

Totally, 138 clinical samples previously confirmed adenovirus-positive were detected by tq-PCR assay. The results indicated that 42 (30.43%) were serotype 2, 40 (28.99%) were serotype 3 and 5 (3.62%) were serotype 7. Besides, two clinical samples were serotype 2,7 and 2,3 co-infections. HAdV serotypes 1 (22,15.94%), 5 (13,9.42%), 6 (6,4.35%), 4 (2,1.45%), 31(1,0.73%), 55 (1,0.73%) and 57 (1,0.73%) were identified in 46 out of 138 samples by the sequencing of nested PCR product. The agreement between the tq-PCR and two step nested PCR was 96.38%(133/138) as showed in Table 5. In the present study with limited sample size, the tq-PCR diagnostic sensitivities of HAdV2, 3, and 7 were found to be 97.7% (42/43), 90.1% (40/44) and 100% (5/5), respectively.

**Table 5:** Agreement between the tq-PCR and two step nested PCR

	Triplex real-time PCR		Two step nest PCR		Agreement
	Positive	Negative	Positive	Negative	
Ad2	42	94	43	93	99.26%
Ad3	40	96	44	92	97.05%
Ad7	5	131	5	131	100%

*Footnote*: of 138 HAdV positive samples were detected by these two methods, as nested PCR can’t show co-infection serotype 2, 7 and 2, 3, so only 136 clinical samples enrolled in agreement between the tq-PCR and two step nested PCR

## Discussion

HAdV2, 3, and 7 were most commonly reported associated with ARI worldwide [3, 16]. Type2 is a common causes of ARI worldwide, but appear to be less virulent than type3 and type7 [17, 18], Type3 is the common serotype implicated in HAdV infections in children and adults [6, 16]. Type7 was the leading common serotype reported association with respiratory illness and it appears to be more virulent than other serotypes that may occur fatal pneumonias in immunocompetent children [7, 19] and adults [15]. In China, HAdV serotypes 2, 3 and 7 were the most prevalent types in pneumonia children [14]. Epidemic outbreaks caused by HAdV2, 3, and 7 affected numerous of populations in Chongqing, Guangzhou [20], Beijing [21], Shanxi [7], Hangzhou [22, 23] and Taiwan [3, 6, 24]. Thus differential detection of HAdV2, 3, and 7 from other serotypes in China should be of great significance in clinical setting. Besides, Species B HAdV types 3 and 7 have a high probability of disease association. In contrast, species C HAdV type 2 is often present as a bystander, as shown in multiple studies that included asymptomatic controls. Whereas types 3 and 7 have epidemic potential, being associated with outbreak clusters of ARI, type 2 and other species C viruses rarely cause outbreaks.

In recent years, traditional PCR method to detect serotypes of adenovirus [25, 26] needs gel electrophoresis and sequencing, which significantly increases the risk of cross-contamination and is also time consuming and labor intensive. Fluorescence quantitative PCR for adenovirus typing showed acceptable sensitivity, specificity, and reproducibility. In previous studies by others [9, 27, 28], the quantitative real-time PCR (panel assay) has proven to be of great value for the differential detection of HAdV types, but it is excessively reagent-consuming and costly. The nested PCR described above [13] seems more sensitive compared with other methods, but it’s too inconvenient to be applicable in clinical laboratories with two-step amplification followed by sequencing. The tq-PCR assay in this study is more convenient and rapid to detect the most common HAdV serotypes 2, 3 and 7 in a single closed tube, thus it is very suitable for HAdV typing to meet clinical diagnosis purpose with advantage of low cost and less time. To our best knowledge, this is the first report on a tq-PCR assay for differential detection of HAdV serotypes 2, 3 and 7.

The tq-PCR revealed stable repeatability and a sensitivity of 100 copies/reaction and no cross reaction with other common respiratory viruses or HAdV serotypes 1,4,5,6,31 and 55. Five samples out of 138 negative by tq-PCR were positive by nested PCR, we speculated that these samples had a low virus titer that is below the detection limit of the tq-PCR. Although tq-PCR is slightly less sensitive compared with previous panel assay and nested PCR, and the efficiency of tq-PCR has not been demonstrated with quantitative results in clinical samples, it had sufficient sensitivity and adequate for the differential diagnosis of HAdV infections. Treatment options for patients with HAdV infection are limited [29]. Generally, when patients suffer from HAdV infection and show a rapid and high increase of viral load in the serum [10, 11], clinical protocols recommended are intensive supportive care and application of antiviral drugs [30], so early specific and differential diagnosis by the use of tq-PCR can be more targeted to ensure prompt treatment, which will be aided in effectively controlling the rapid replication of the HAdV in the body.

The HAdV spectrum associated with ARI is broad in China. In previous studies, HAdV types 1,4,5,6,14,55 [24, 31, 32] were also reported to be associated with ARI. However, HAdV serotypes 2, 3 and 7 were more frequently reported to cause outbreaks and ARI. In our study, HAdV serotypes 2, 3 and 7 were most prevalent (63.4%, 83/138) in Hebei, China between June and November 2017, which is consistent with previous reports in China.

## Conclusions

In conclusion, we established a tq-PCR assay with appropriate sensitivity, high specificity and reproducibility. tq-PCR assay offers the advantages of rapid detection, cost-effectiveness, and convenience and allows simultaneous and differential detection of HAdV serotypes 2, 3, and 7, which might be of great potential for clinical use.

## Abbreviations

ARI: Acute respiratory infections
CV: Coefficient of variation
HAdV: human adenovirus
PCR: Polymerase Chain Reaction
tq-PCR: Triplex quantitative real-time PCR assay
